# Analysis of clinical characteristics and risk factors of recurrent *Clostridioides difficile* infection in children

**DOI:** 10.3389/fped.2025.1571199

**Published:** 2025-07-18

**Authors:** Jin-Yue Huang, Ning Wang, Jing-Yao Wang, Hua-Chu Zuo, Juan Li, Yu Zhao

**Affiliations:** ^1^ Tianjin Pediatric Research Institute, Tianjin Key Laboratory of Birth Defects for Prevention and Treatment, Tianjin Children's Hospital (Children's Hospital of Tianjin University), Tianjin, China; ^2^ Department of Gastroenterology, Tianjin Children's Hospital (Children's Hospital of Tianjin University), Tianjin, China; ^3^ Department of Pathology, Tianjin Children's Hospital (Children's Hospital of Tianjin University), Tianjin, China

**Keywords:** children, *Clostridioides difficile* infection, recurrence, clinical characteristics, risk factors

## Abstract

**Objective:**

To analyze clinical characteristics and risk factors for recurrence of *Clostridioides difficile* infection (CDI) in children.

**Methods:**

Clinical data were retrospectively collected from all children (<18 years) hospitalized with CDI at Tianjin Children's Hospital between September 2018 and December 2023.

**Results:**

In total, 115 patients (66 males and 49 females;) were divided into recurrence (*n* = 38) and non-recurrence (*n* = 77) groups. Logistic regression was used to compare clinical data and analyze risk factors for CDI recurrence. The recurrent CDI(rCDI) and non-recurrence groups had statistically significant differences in terms of age, comorbidities, prior antibiotic exposure, mode of CDI acquisition (hospital vs. community), colonoscopy(the rationale for colonoscopy data inclusion—diagnosis confirmation or ruling out other causes), treatment selection, levels of interleukin-6 and creatine kinase, and body mass index (*p* < 0.05). Multivariate analysis showed that healthcare-associated CDI [odds ratio [OR] = 14.754, 95% confidence interval [*CI*]:4.568–47.650, *p* = 0.000] is an independent risk factor for CDI recurrence in children (*p* < 0.05), with sensitivity and specificity of 73.7% and 87.0%, respectively.

**Conclusion:**

Healthcare-associated CDI (HA-CDI)is an independent risk factor for pediatric rCDI, and the introduction of this indicator in diagnosis has certain accuracy in predicting rCDI.

## 1 Introduction

*Clostridioides difficile* (CD) is a Gram-positive anaerobic bacillus first isolated from the feces of healthy newborns by Hall and Toole in 1935. It is classified as an opportunistic pathogen (1). *Clostridioides difficile* infection (CDI) typically occurs in patients undergoing prolonged treatment with antibiotics or acid suppressants, and the clinical severity can vary significantly. While mild cases may present with asymptomatic colonization or mild diarrhea, severe cases can lead to severe colitis, toxic megacolon, sepsis, shock, and even death (2). Recent domestic and international studies have shown that the extensive spread of highly virulent strains (NAP1/BI/027) has led to a significant increase in the incidence of CDI and the recurrence rate of infection.CDI is a leading cause of hospital-acquired intestinal infections and antibiotic-associated diarrhea, contributing to increased healthcare costs and mortality. Vancomycin and metronidazole are the first-line treatments for CDI. Fecal microbiota transplantation (FMT) is becoming part of the treatment algorithms against rCDI both in adult and pediatric gastroenterology practice (3). The rate of rCDI in children after initial treatment is as high as 20%–30% (4, 5). Previous studies have shown that the risk factors for rCDI mainly include antibiotic use, proton pump inhibitors, chemotherapy, immunosuppressive drugs, previous hospitalizations, recent gastrointestinal surgeries, and underlying diseases, such as inflammatory bowel disease or the presence of tracheostomy or gastrostomy tubes, etc (6). Unfortunately, the mechanism of rCDI is still unclear, and the research on the risk factors of rCDI in children is still relatively limited, which brings significant challenges to clinical management. This study aims to investigate the clinical characteristics and risk factors of rCDI in children to enable early identification, diagnosis, and treatment of rCDI.

## 2 Materials and methods

### 2.1 Study design statement

A single-center, retrospective study, was conducted between September 2018 and December 2023 in which electronic medical records were searched for a diagnosis of CDI in all children hospitalized with CDI in the Department of Internal Medicine, Tianjin Children's Hospital. Only children (age <18 years) who had a first confirmed CDI meeting the diagnostic criteria for pediatric CDI (7) could be included. The study procedure was approved by the ethics committee of the Tianjin Children's Hospital (2024-LXKY-009). Patients with incomplete clinical or laboratory data, such as routine bacterial culture and virus testing in feces, were excluded.

The criteria to meet the diagnosis of CDI were based on the Clinical Practice Guidelines for CDI in Adults and Children published by the Infectious Diseases Society of America and the Society for Healthcare Epidemiology of America in 2017(The following text will all be referred to as the guidelines) (7). CDI is defined by the presence of gastrointestinal symptoms (diarrhea, increased frequency of bowel movements, bloody stools, intestinal spasms, and/or urgency) combined with one of the following criteria: (1) positive for CD glutamate dehydrogenase (GDH) and positive for CD toxin A or B by enzyme-linked immunosorbent assay; (2) positive for CD GDH, negative for CD toxin A and B by enzyme-linked immunosorbent assay, and positive for CD by real-time fluorescence PCR; and/or (3) colonoscopy or histopathology showing pseudomembranous enteritis.

Recurrence is determined by the reappearance of symptoms and positive CD fecal assay result within 8 weeks after antibiotic treatment for a confirmed CDI. In addition, CDI can be acquired in health care settings or in the community. Healthcare-associated CDI(HA-CDI) refers to the appearance of clinical manifestations and symptoms occurring ≥72 h after admission or <72 h after admission if patient was previous hospitalized within the previous 4 weeks after discharge. Community-associated (CA-CDI) refers to infections occurring within ≤72 h after hospital admission and without a history of exposure to healthcare facilities within 12 weeks prior to admission (8).

### 2.2 Research inclusion and exclusion process

**Figure 1:**
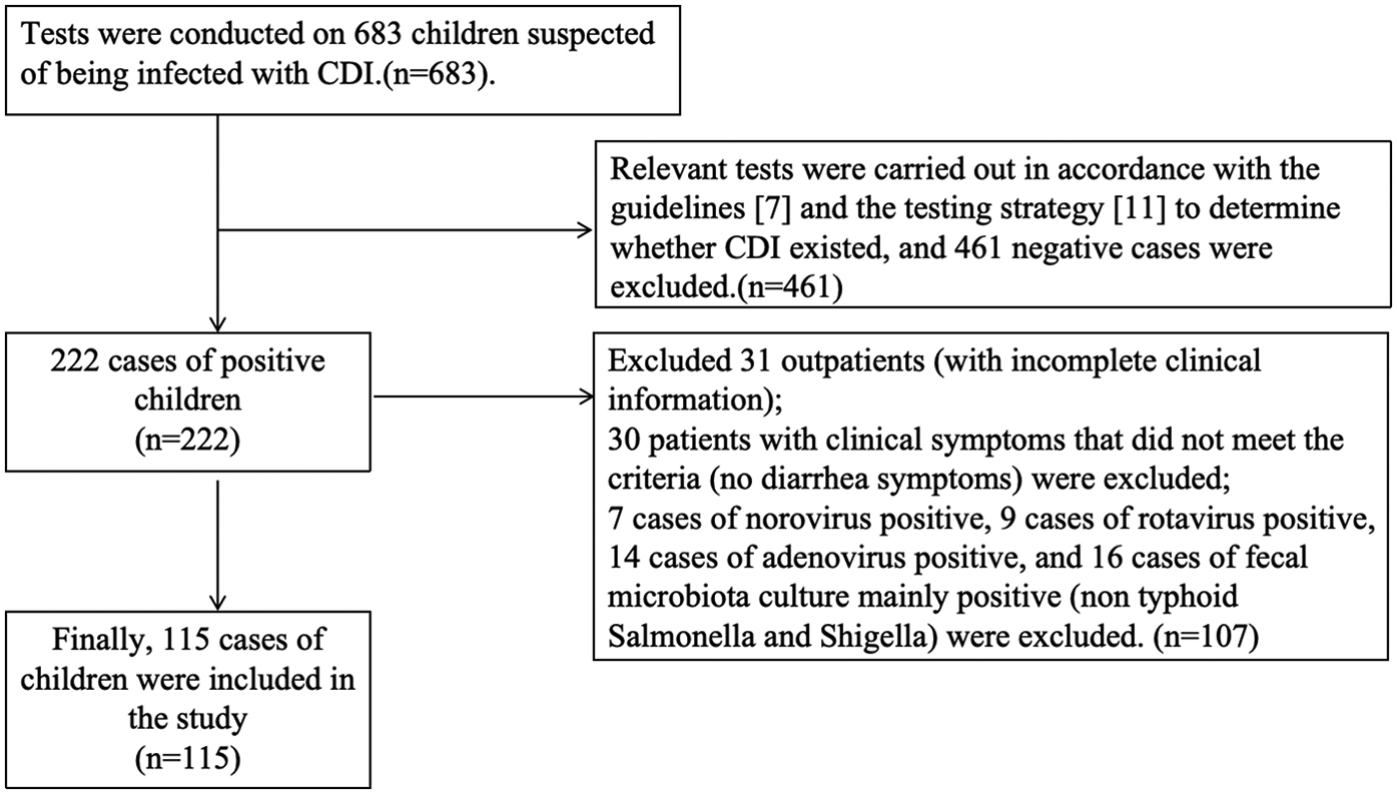
Shows the inclusion and exclusion process and results of the study.

Relevant tests were carried out in accordance with the guidelines (7) and the testing strategy (9) to determine whether CDI existed Figure 1.

### 2.3 Data collection

General clinical data of CDI patients were collected through the hospital's electronic medical record system. The data included gender, age at admission, presence of comorbidities (Including: cancer and chemotherapy; patients on immunosuppressives or autoimmune conditions on immunosuppressives; The use of proton pump inhibitors; The presence of tracheostomy or gastrostomy tubes; Other gastrointestinal diseases, congenital heart disease, genetic metabolic diseases, etc), CDI-related signs and symptoms, laboratory and auxiliary examinations, prior antibiotic use, surgical history within the past 6 months, whether colonoscopy was performed[the rationale for colonoscopy data inclusion—diagnosis confirmation or ruling out other causes, e.g., inflammatory bowel disease(IBD), polyps, perianal lesions or other systemic diseases involving the lower digestive tract, etc] and mode of CDI acquisition (HA-CDI/CA-CDI).

### 2.4 Statistical analysis

Statistical analyses were performed using SPSS 26.0 statistical software. Normally distributed data were represented by mean ± standard deviation, and inter-group comparisons were conducted using the t-test and one-way analysis of variance. Non-normally distributed data were represented by median (P_25_,P_75_), and inter-group comparisons were performed using the Wilcoxon rank-sum test and the Kruskal–Wallis test. Categorical data were represented by frequency or percentage, and inter-group comparisons were made using the *χ*² test. Multivariate logistic regression analysis was employed to explore the risk factors for rCDI. Variables with an inclusion level of 0.05 and an exclusion level of 0.10 were screened. The odds ratio (OR) and its 95% confidence interval (*CI*) were calculated. A *P*-value < 0.05 was considered statistically significant.

## 3 Results

### 3.1 Clinical characteristics of patients

A total of 115 patients were included in this study. Among these patients, 62 cases (53.91%) were diagnosed with persistent diarrhea, 26 cases (22.61%) presented with hemorrhagic colitis, 22 cases (19.13%) had known or subsequently diagnosed IBD(of which 16 cases were already diagnosed at the time of inclusion in this study and were in remission according to disease activity assessment; 6 patients were diagnosed at the time of inclusion in this study, without excluding the possibility of their own disease due to clinical symptoms), 4 cases (3.48%) had juvenile polyps(in addition to polyps, all four patients had manifestations of colitis under colonoscopy), and another four cases (3.48%) had genetic diseases(previously diagnosed as 2 cases of methylmalonic acidemia, 1 case of Joubert syndrome, and 1 case of Noonan syndrome, these four patients had feeding difficulties due to underlying diseases, but none of them had nasogastric tube or gastric stoma surgery, with a certain degree of nutritional difficulties). One case (0.87%) involved intussusception, which required emergency surgical treatment(the patient, previously in good health, was initially admitted to the hospital due to acute colitis. Severe diarrhea subsequently led to intussusception, necessitating emergency surgery). A total of 76 cases (66.09%) had a history of antibiotic exposure within 30 days prior to admission. The main clinical manifestations of CDI in these patients included hematochezia (70.43%), abdominal pain (37.39%), fever (32.17%), and vomiting (14.78%). Among the newly diagnosed children with CDI, the incidence of CA-CDI was higher (66.96%) compared to HA-CDI (33.04%). Regarding treatment plans, 47 patients (40.87%) received metronidazole, while 68 patients (59.13%) were treated with a combination of vancomycin and other drugs (Other medications are energy supplements for adjuvant therapy, such as vitamin C and adenosine triphosphate). Supplementary note: fidaxomicin has not yet been launched in the Chinese market, and FMT has not been carried out in this medical institution at present. Therefore, fidaxomicin and FMT were not involved in the treatment of all cases in this study.

### 3.2 Group comparison of basic information and clinical characteristics

In this study, 38 cases (33.04%) experienced recurrence after treatment and were categorized as the recurrent group, 17 patients had ≥2 CDI recurrences, while 77 cases (66.96%) did not experience recurrence and were categorized as the non-recurrent group. There were statistically significant differences (*p* < 0.05) between the recurrent and non-recurrent groups in terms of age, the presence of comorbidities, history of antibiotic exposure, mode of CDI acquisition, colonoscopy examination, treatment plan selection, levels of interleukin (IL)-6 and creatine kinase (CK), and body mass index. Other indicators did not show statistically significant differences (*p* > 0.05), as shown in Table 1.

**Table 1:** Comparison of clinical characteristics between recurrent and non recurrent groups [(x ± s), M (P25, P75), *n* (%)].

Variables	Recurrent (38)	Non recurrent(77)	*Χ*^2^/Z	*P*
Gender (Male/Female)	20/18	46/31	1.171	0.279
Age [M (P_25_,P_75_), m]	48.0 (12.00, 114.00)	12.0 (11.00, 72.00)	−2.098	0.036
Symptoms				
Fever	12 (31.58%)	25 (32.47%)	0.880	0.348
Abdominal distension	2 (5.26%)	4 (5.19%)	0.021	0.912
Abdominal pain	16 (42.11%)	27 (35.06%)	1.114	0.291
Vomiting	5 (13.16%)	12 (15.58%)	0.033	0.855
Hematochezia	26 (68.42%)	55 (71.43%)	0.080	0.777
Other related medical history				
History of antibiotic exposure	24 (63.16%)	52 (67.53%)	10.743	0.001
History of comorbidities	23 (60.53%)	22 (28.57%)	13.487	0.000
History of lactose intolerance	17 (44.74%)	35 (45.46%)	0.031	0.861
History of allergic rhinitis	6 (15.79%)	6 (7.79%)	2.178	0.140
History of eczema	15 (39.47%)	39 (50.65%)	0.589	0.443
Laboratory index				
WBC(4.40–11.90 × 10^12 ^/L)	9.96 (7.30, 12.90)	9.97 (7.75, 12.87)	−0.553	0.580
CRP(0.00–8.00 mg/L)	2.50 (2.50, 3.79)	2.50 (2.50, 3.54）	−0.167	0.867
PCT(0–0.05 ng/ml)	0.06 (0.03, 0.08)	0.07 (0.04, 0.13)	−1.385	0.166
IL-6 (0.00–7.00 pg/ml)	6.42 (1.66, 12.07)	2.01 (1.50, 4.74)	-2.496	0.013
ALB(39.0–54.0 g/L)	44.40 (41.10, 46.00)	44.70 (42.70, 46.70)	-0.926	0.354
Scr(19–44 umol/L)	31.5 (23.0, 44.75)	27 (21.00, 36.00)	-1.702	0.089
GLU(3.90–6.10 mmol/L)	2.70 (2.10, 3.60)	4.89 (4.31, 5.46)	-0.344	0.731
TP(61.0–79.0 g/L)	64.20 (60.30, 68.50)	65.50 (61.95, 68.10)	-0.223	0.824
AST(14–44 U/L)	29.00 (20.00, 40.00)	33.00 (25.00, 44.75)	−1.702	0.089
CK(40–200 U/L)	91.00 (40.50, 118.25)	107.00 (74.00, 149.00)	−1.977	0.048
CK-MB(0–24 U/L)	12.00 (5.25, 23.25)	11.00 (6.11, 17.50)	−0.439	0.660
BMI (18–24 kg/m^2^)	16.11 (14.08, 17.33)	15.70 (14.41, 17.35)	−2.351	0.019
Mode of CDI acquisition				
CA-CDI	2 (5.26%)	73 (94.81%)	97.561	0.000
HA-CDI	36 (94.74%)	4 (5.19%)		
Colonoscopy examination	22 (57.89%)	20 (25.97%)	13.668	0.000
Treatment methods				
Metronidazole	27 (71.05%)	21 (27.27%)	21.316	0.000
Vancomycin/combination with other antibiotics	11(28.95%)	56(72.73%)		

WBC, white blood cells; CRP, C-reactive protein; PCT, Procalcitonin (The rational for PCT data inclusion is for assisting in the early diagnosis and evaluation of bacterial infections, sepsis, and severe infections, guiding antibiotic use, and monitoring treatment efficacy); IL-6, interleukin-6 (The rational for IL-6 data inclusion is a marker of early increase of inflammatory response, which is helpful for the diagnosis and differential diagnosis of infectious diseases, can reflect the activity of inflammation and immune response in the body, and can be used to monitor the progress and treatment effect of inflammatory diseases); ALB, albumin; Scr, serum creatinine; GLU, glucose; TP, total protein (The rational for TP data inclusion is an important indicator for evaluating protein metabolism and health in the body, mainly used to reflect nutritional status, chronic disease consumption, or immune abnormalities. It can be used as an important indicator for evaluating malnutrition, absorption disorders, or metabolic abnormalities); AST, aspartate aminotransferase; CK, creatine kinase; CK-MB, creatine kinase MB isoenzyme; BMI, body mass index.

### 3.3 Logistic regression analysis for rCDI in children

After univariate analysis, variables with statistical significance (*p* < 0.05) between the recurrent and non-recurrent groups were included in the multivariate logistic regression analysis. Nine variables were included as independent variables: age, comorbidities, history of antibiotic exposure, mode of CDI acquisition, colonoscopy examination, treatment plan selection, IL-6 levels, CK levels, and BMI values. The dependent variable was whether the child experienced rCDI. Multivariate logistic regression analysis revealed that HA-CDI was an independent risk factor for rCDI in children (*p* < 0.05), as shown in Table 2.

**Table 2:** Logistic regression analysis of influencing factors in children with RCDI.

Variables	B	SE	Wald	*P*	OR	95%CI
Age	0.004	0.007	0.316	0.574	1.004	0.990–1.018
History of antibiotic exposure	1.744	0.946	3.396	0.065	5.719	0.089–36.542
Treatment methods	−0.208	0.620	0.113	0.737	0.812	0.241–2.737
Mode of CDI acquisition	2.691	0.598	20.245	0.000	14.754	4.568–47.650
History of comorbidities	0.261	0.747	0.122	0.727	1.298	0.300–5.613
Colonoscopy examination	0.285	0.688	0.172	0.678	1.330	0.345–5.120
CK	0.002	0.006	0.200	0.655	1.003	0.992–1.014
IL-6	0.016	0.012	1.802	0.180	1.016	0.993–1.041
BMI	−0.181	0.134	1.842	0.175	0.834	0.642–1.084
Constant	−1.118	2.274	0.242	0.623	0.327	

### 3.4 Receiver operating characteristic curve (ROC) curve evaluation for the accuracy of rCDI diagnosis

**Figure 2:**
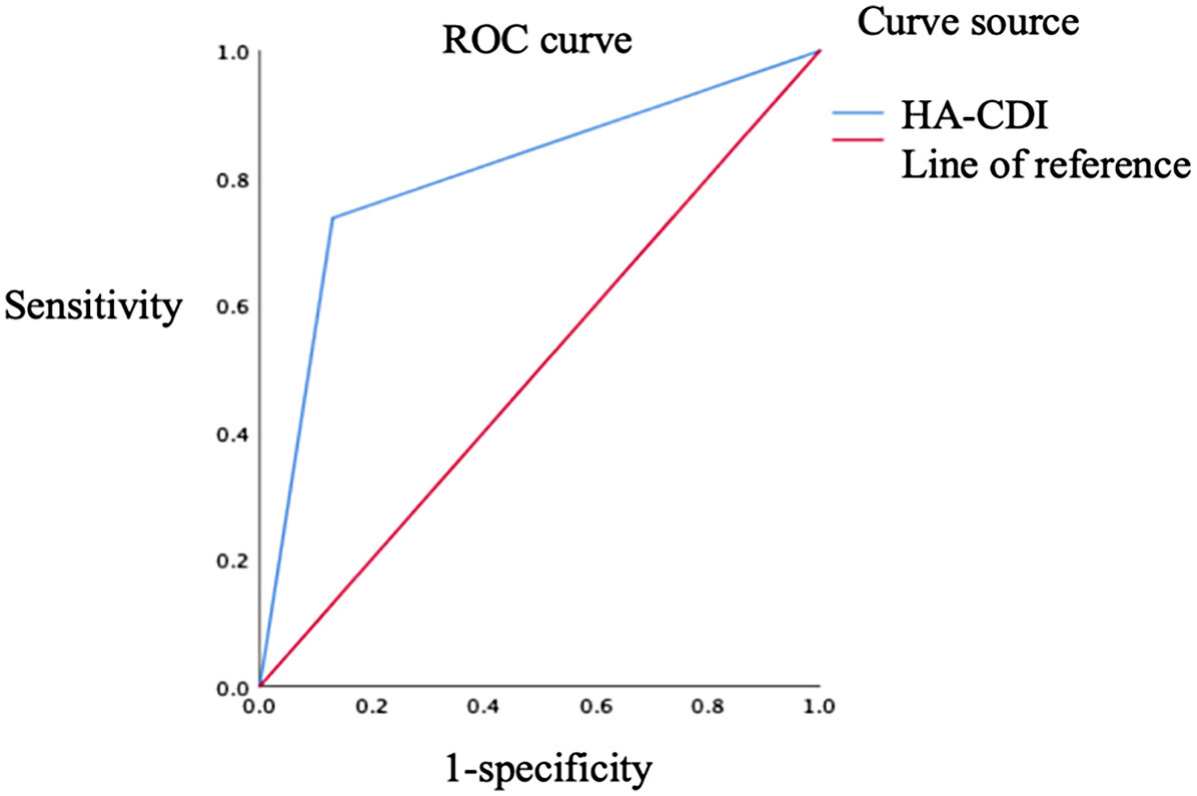
ROC curve of HA-CDI diagnostic prediction for rCDI.

Figure 2 shows that when the cut-off of HA-CDI is ≥0.5, the area under the ROC curve is 0.803 and the standard error is 0.048, *P* < 0.001, 95%*CI*:0.710–0.897, The sensitivity and specificity of HA-CDI in predicting rCDI were 73.7% and 87.0%, respectively, with a Youden's Index(YI) of 0.607, indicating a certain diagnostic and predictive effect Figure 2.

## 4 Discussion

Previous studies have mainly focused on the risk factors for rCDI in adult patients, particularly on highly virulent strains, advanced age, and exposure to antibiotics and acid suppressants (5). Research on rCDI in children is relatively limited. The present study demonstrated a CDI recurrence rate of 31.30%, which is similar to the upper limit of epidemiological data on rCDI in foreign children (10). 66.09% of the children in this study had a history of antibiotic exposure before the diagnosis of CDI. In the one-way analysis of variance on recurrence, it was found that there was a statistically significant difference in recent antibiotic exposure history between the recurrence group and the non recurrence group. The possible reason is that antibiotics disrupt the natural gut microbiota, creating favorable conditions for CD colonization and reproduction. There is a certain degree of empirical and widespread use of broad-spectrum antibiotics in China, which increases the risk of CDI occurrence and recurrence in children. Remind clinical physicians to use antibiotics comprehensively and reasonably. Medical staff should maintain proper hand hygiene after coming into contact with pediatric patients. However, traditional risk factors do not always exist. In another multi site case-control study targeting young children with rCDI, 13.6% of the children lacked any identifiable risk factors (11), which also suggests that medical researchers need to further strengthen and refine their research on pediatric rCDI.

There are studies suggesting that risk factors for rCDI include the presence of complications (such as malignant tumors, inflammatory bowel disease), recent surgeries, etc (5). In this study, various factors were analyzed among CDI patients, including comorbidities (eg: cancer and chemotherapy; patients on immunosuppressives or autoimmune conditions on immunosuppressives; The use of proton pump inhibitors; The presence of tracheostomy or gastrostomy tubes; Other gastrointestinal diseases, congenital heart disease, genetic diseases, etc), allergic-related diseases (such as eczema, allergic rhinitis, and food protein allergies), Ig classification and quantitative levels, common laboratory auxiliary tests, and other indicators. There were statistical differences between recurrent group and non recurrent group in complications, IL-6 and CK levels, BMI and HA-CDI incidence rate (*p* < 0.05). Genetic diseases include 2 cases of methylmalonic acidemia, 1 case of Joubert syndrome, and 1 case of Noonan syndrome, all of which exhibit neurological involvement such as feeding difficulties, developmental delays, and intellectual disabilities. This suggests that attention should be paid to these diseases in the diagnosis and treatment of rCDI. However, due to sample size limitations and selection bias in research design, these comorbidities need to be further validated as risk factors by expanding the sample size. In addition, compared with asymptomatic carriers and the control group, patients with CD related diarrhea have significantly higher levels of anti surface protein IgG (12). However, this study did not find a correlation between Ig classification, quantification, and rCDI in children. At present, there is no recommendation in the guidelines (7) to use biomarkers as auxiliary diagnosis. Some scholars have evaluated 17 biomarkers in the serum of patients with CD colitis, and logistic regression analysis shows that, IL-6, Procalcitonin, IL-8, IL-2R, and hepatocyte growth factor are significantly correlated with adverse outcomes of CDI (13), but the validation of biomarkers related to rCDI was still relatively lacking. This study found a correlation between IL-6 and rCDI, however, the limited sample size and single center retrospective design in this study introduce inherent selection bias, especially in the classification of HA-CDI and CA-CDI. We plan to further expand the sample size and strengthen multi center research in the future to explore the exact role of new quantitative indicators in CDI and rCDI.

The findings revealed that HA-CDI was an independent risk factor for rCDI in children (*p* < 0.05). Receiver operating characteristic curve analysis indicated that HA-CDI exhibited a sensitivity and specificity of 73.7% and 87.0%, respectively, in predicting rCDI. A retrospective study by Nicholson et al. (14) on 186 children with CDI identified recent hospitalization as a risk factor for rCDI, with HA-CDI patients exhibiting more severe conditions and a higher proportion of combined antibiotic use. Similarly, other retrospective studies on adult CDI recurrence have shown that HA-CDI is associated with the occurrence of rCDI (15, 16). These research results are consistent with the findings of this study, indicating that the main factor leading to pediatric rCDI is HA-CDI, and this multivariate analysis will correct multiple tests to avoid incorrect statements. Therefore, HA-CDI has a certain sensitivity and specificity in predicting rCDI, However, as HA-CDI is a binary variable, although it can be used as a test variable, due to its only two values (such as 0/1), the generated ROC curve may only contain 1–2 valid data points and cannot fully reflect the sensitivity and specificity changes under different thresholds.

This study is a single center retrospective analysis with inherent selection bias in its design; The limited number of cases included in the study, especially the small sample size of the recurrence group, may limit the detection of significant associations such as comorbidities and IL-6 levels; The included cases in the study are those who are under 1 year old and have uncertain diagnoses (such as IBD, polyps, intussusception, etc.), which may also lead to selection bias. In addition, the predictive efficacy of HA-CDI proposed in this study as a predictor of rCDI in children needs further validation. Especially for young children with asymptomatic CD colonization, the diagnosis and prediction of such patients are still limited. The diagnostic process relies on previous guidelines, and future research will continue to expand the sample and multi center studies, prioritizing continuous or ordered categorical variables as test variables, in order to comprehensively evaluate model performance by adjusting thresholds. Due to limitations in sample size and current laboratory data testing techniques, future research should focus on multicenter, large sample, prospective randomized controlled trials and explore new immunological quantitative indicators to further identify risk factors for childhood rCDI and optimize predictive values.

## 5 Conclusion

This study investigates the clinical characteristics and risk factors of rCDI in children, identifying HA-CDI as an independent risk factor, based on this indicator, has certain sensitivity and specificity for predicting rCDI.

## Data Availability

The original contributions presented in the study are included in the article/Supplementary Material, further inquiries can be directed to the corresponding authors.
